# Drastic Reduction Inpatient Visits to the Emergency Department in a Hospital in Israel During the COVID-19 Outbreak, Compared to the H1N1 2009

**DOI:** 10.34172/ijhpm.2020.151

**Published:** 2020-08-09

**Authors:** Fuad Basis, Hisam Zeidani, Khetam Hussein, Shlomo Hareli

**Affiliations:** ^1^Technion Faculty of Medicine, Rambam Health Care Campus, Haifa, Israel.; ^2^Haifa University, Haifa, Israel.

**Keywords:** COVID-19, H1N1 Influenza Pandemic, Panic, Patients, Emergency Hospital Service

## Abstract

**Background**: On February 26, 2020, the first case of coronavirus disease 2019 (COVID-19) was detected in Israel. The Ministry of Health (MoH) instructed people to take isolation measures and restrict their movement. Similarly, there was a gradual decrease in the number of visits to our emergency department (ED).

**Objectives**: To describe the decline in the referrals to the ED and in-hospital beds occupancy during the COVID-19 pandemic and to compare it to the H1N1 2009 pandemic.

**Methods**: Employing a cross-sectional epidemiologic study, the pattern of visits to the ED during the COVID-19 was compared with the pattern of visits during the 2009 H1N1 pandemic, as well as a year without a pandemic. The data was adjusted to consider changes in population size. The Welch t test for unpaired, unequal samples was used to analyze the data.

**Results**: Within 2 months of the COVID-19 outbreak, the average number of visits to the ED dropped by 30.2% and the hospital occupancy by 29.2% (a minimum of 57%), compared to the same period, the year before. In comparison to the same period during the H1N1 outbreak, we witnessed a significant decline in the number of visits to the ED during the COVID-19 outbreak.

**Conclusion**: The behavior of people during the COVID-19 pandemic was different from their behavior during the H1N1 pandemic. People seemed to avoid visiting the ED. The boundary between precaution and panic in the generation of the media could be very thin. Decision-makers must take this into account.

## Background

 Like in many other countries, Israeli hospitals are usually crowded during winter seasons, and the average occupancy in our hospital is 98.5% of the hospital beds, in the last 5 years. The winter of 2020 (from October 1, 2019, until April 30, 2020) seemed to be different in relation to the population’s attitude and behavior regarding the fear of becoming sick, due to the outbreak of the coronavirus disease 2019 (COVID-19) pandemic, in comparison with the same period the year before. This led to the question of whether this pattern of behavior was typical of pandemics as such or whether it was unique to the COVID-19 pandemic.

 Normally, during the winter seasons in most western countries, the emergency departments (EDs) become very crowded. In extreme situations, ambulances move patients away from crowded to less crowded EDs in many hospitals across the world.^1,2^ This situation is reflected well by statistics published by the Israeli Ministry of Health (MoH) in January 2011. The statistics showed that only 278 of the 787 patients on mechanical ventilation were kept in the intensive care units. The rest of the patients had to be mechanically ventilated in the general medicine wards.^3^However, this winter after the COVID-19 outbreak at the end of February 2020, we witnessed a gradual decline in the hospital occupancy by 29.2% on average (a minimum of 57%).

 Seasonal influenza is not the only reason that can change the occupancy in hospitals. The outbreak of pandemics, some of which occur during the winter, also contributes to hospital occupancy. This was the case with the severe acute respiratory syndrome(SARS) outbreak in November 2002.^4^ However, some pandemics may erupt during other periods of the year. This was the case with H1N1 pandemic which started after the winter, was first described in April 2009,^5^ and was detected in Israel on April 30, 2009.

 This year, a new epidemic erupted in the world, and the first COVID-19 virus case in Israel was reported on February 23, 2020. Shortly thereafter, at the beginning of March 2020, the MoH came with new instructions concerning citizens’ isolation and traffic restrictions. After these restrictions, from March 1 until April 30, 2020 (the study period), the hospital’s occupancy dropped gradually to a minimum of 57% (29.2% in average), in comparison to 98.25% in the same period in 2009 during the H1N1 pandemic. In our hospital, like in other hospitals in the country, all the ambulatory procedures were cancelled. However, we did not witness this pattern of policy during the SARS and the H1N1 pandemics.

## Objectives

 This study aimed to describe the decline in the referrals to the ED and in-hospital beds occupancy during the COVID-19 pandemic and to compare it to referrals during the H1N1 2009 pandemic.

## Materials and Methods

 This was a cross-sectional epidemiologic study. All the data was collected from the business intelligence computerized system. We collected the average number of visits to the ED, on weekly basis (how many visits to the ED were made each week), from October 1, 2019, until February 28, 2020 (during the seasonal influenza period 2019-2020), and we compared it to the average weekly visits during the COVID-19 outbreak (from March 1 until April 30, 2020).

 The first case of H1N1 was detected in Israel toward the end of April 2009. To check if there was a difference in the average weekly visits during the H1N1 outbreak, in comparison to the same period a year before, we compared the number of visits to the ED from May 1 until December 31, 2009, every week, to the same period a year before (2008).

**Figure 1:**
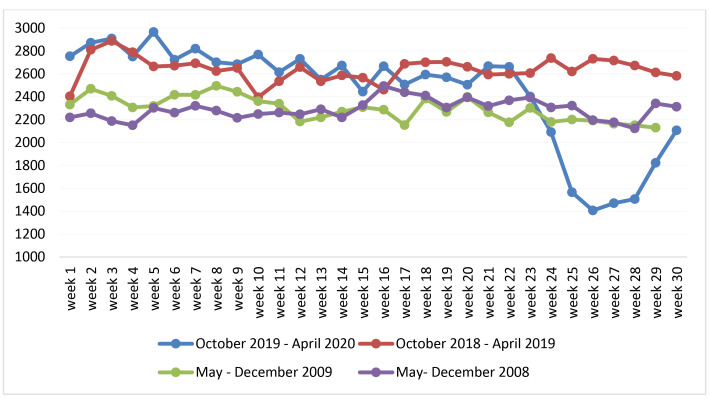
The Number of Weekly Visits to the Emergency Department Starting From the First Week of the H1N1 and COVID-19 Outbreaks, in Comparison to the Previous Year. Abbreviation: COVID-19, coronavirus disease 2019.

 The H1N1 virus is a variant of the flu viruses. However, the H1N1 outbreak started in springtime, while the seasonal flu starts in the northern part of the globe at wintertime. Therefore, we had to compare the number of visits to our ED during the seasonal flu before the COVID-19 outbreak (from October 1, 2019 until February 28, 2020) to the number of visits to the ED during the H1N1 outbreak (from May 1 until December 31, 2009). Since we compared different months and periods, we chose to present all periods comparison as a function of the weeks starting with the first week of the pandemic (week 1, week 2… etc) (Figure 1).

 Because the data concerned the number of visits to the ED during different periods, separated by 11 years, we adjusted the number of visits such that they were represented as the number of visits per week as a function of relevant population size in the respective year. The population of Haifa, which forms the potential pull of patients, increased from 263 477 in 2009 to 283 405 in 2019 (7.5% increase).^6^ The adjustment was calculated by dividing the number of visits per week by the number of residents of Haifa in the respective year (changes in population size by week or month were not available).

 Finally, we wanted to check the number of deaths from the COVID-19 among admitted patients, in comparison to the number of deaths from seasonal flu complications. We thought it would be interesting to see if the rate of deaths in our hospital was much higher than that during the seasonal flu. To do that, they compared data from the infections control unit about the number of deaths, due to seasonal influenza, to that of the COVID-19 pandemic. This analysis employed the Fisher test for small populations.

**Figure 2:**
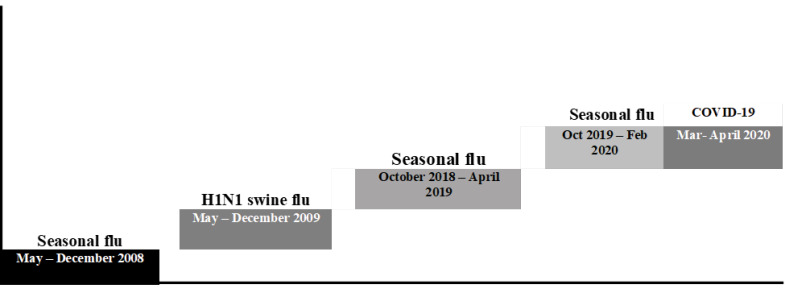
The Timeline of the Periods Under Study. Abbreviation: COVID-19, coronavirus disease 2019.

 A series of Welch’s independent *t* tests were used to test the degree of the average number of visits to the ED weekly, which varied between the periods of interest. The Welch’s *t* test was used for the unequal number of data points across the periods of interest. Reported degrees of freedom were rounded up to the nearest integer. To follow the sequence of the periods under the study, see Figure 2.

## Results

 During 2 months after the outbreak of the COVID-19 pandemic (March 1 until April 30, 2020), there was a 30.2% drop in the average number of visits per month to the ED, in comparison to the average number of visits to the ED from October 1, 2019 until February 28, 2020. All analyses below were performed on the proportion of weekly visits relative to population size during the relevant period. Presented here is the mean number of weekly visits per 100 000. The analysis then showed that the average number of visits to the ED during the COVID-19 was significantly lower (M = 668.97; SD [standard deviation] = 149.52) than that observed during the period before the outbreak (winter seasonal flu October 1, 2019 until February 28, 2020) (M = 949.04; SD = 47.97), *t* (10) = 5.78, *P*< .001. The average number of weekly visits during the COVID-19 was also lower than the average number of weekly visits, during the seasonal flu in the previous year (October 1, 2018-April 30, 2019) (M = 933.54; SD = 40.22), *t* (9) = 5.53, *P*< .001. By contrast, the average number of weekly visits before the outbreak of the COVID-19, during the seasonal influenza period (October 1, 2019, until February 28, 2020), was not significantly different from the average number of weekly visits during the seasonal influenza in the same period in the previous year, *t *(38) = 1.22, *P* = .231.

**Figure 3:**
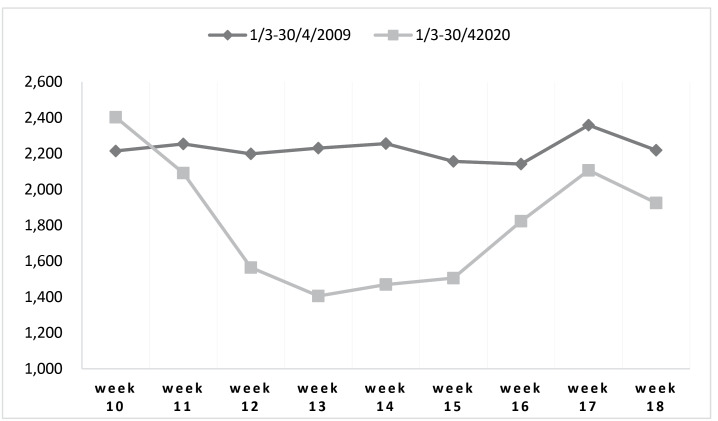
The Number of Visits to the Emergency Department During the COVID-19 Period (March 1 Until April 30, 2020) in Comparison to the Same Period in 2009 (H1N1). Abbreviation: COVID-19, coronavirus disease 2019.

 The average number of weekly visits during the outbreak of H1N1 in 2009 (M = 865.89; SD = 40.08) was not different from that during the same period in the year before (M = 866.35; SD = 32.62), *t* (54) = 0.05, *P*= .958. Finally, the number of visits to the ED during the COVID-19 (March 1 until April 30, 2020) was significantly lower than that during the same period on the H1N1 pandemic 2009, *t* (9) = -4.11, *P*< .002, (see Figure 3). However, the number of visits during the N1H1 was lower than the number of visits during the period before the outbreak of COVID-19 (October 1, 2019, until February 28, 2020, the seasonal flu) (*t* (38) = 6.47, *P*< .001).

 The overall results indicated that during the COVID-19, there was a significant decrease in the number of visits to the ED, relative to the period before it. Also, this decrease in the number of visits brought about a significantly lower level than that observed during the same H1N1 period, which was also slightly lower than that observed during the seasonal flu before the outbreak of COVID-19 (see Figure 1).

 From March 1 until April 30, 2020, during the COVID-19 outbreak, there was a significant decline in the number of visits to the ED, adjusted to change in the population number in comparison to the same period on 2009, *P*< .001 (see Figure 3).

 Data from our hospital revealed that from October 1, 2019, until February 28, 2020, there were 350 admissions due to seasonal influenza complications, thirteen of them died (3.7%). From March 1 until April 30, 2020, there were 90 admissions due to the COVID-19 infection complications, 4 of them died (4.4%). Comparing the number of deaths due to seasonal influenza complications to that from the COVID-19, using the Fisher test, showed that the difference is non-significant (*P*= .756).

## Discussion

 In contrast to other reports about an increase in the number of visits during the H1N1 pandemic,^7-9^ and in comparison, to H1N1 outbreak in Israel, the results of this study make it clear that there was a major change in people’s behavior with regard to EDs visits during COVID-19 pandemic.

 The H1N1 pandemic also caused panic and fear of the unknown. In Australia, for example, children and young adults were more susceptible to H1N1 infection than elderly people, as opposed to the current pandemic.^10^ Isaacs David succinctly described the panic at the beginning of the H1N1 outbreak when he wrote: “The Australian planned response to pandemic influenza including talk of border protection, which had a rather military approach reminiscent of a response to a terrorist attack.”^11^ There was also a panic worldwide as reported by Srinivasan in his article: “Swine flu: is panic the key to successful modern health policy?”^12^ The World Health Organization (WHO) raised the alert level from 3 to 4, which is 2 steps short of declaring a full pandemic.^13^ Despite the fear and panic mentioned above, the number of visits to the ED did not decrease, as it was the case during our study period.

 During the entire period of the COVID-19 outbreak, the coverage of the pandemic occupied the most hours of the day in the news. The news included a constant report of the number of casualties across the world, number of people who were found sick, a discussion of the unpredictable nature as well as the consequences of this virus, and the lack of an effective treatment for it. This was not the case during the H1N1 pandemic. Furthermore, endless reports described the virus as highly contagious, more so than any other virus. News conferences by the heads of the MoH in Israel included hurrying statistics about more than one million infections with more than 15 000 deaths. Also, citizens were advised to refer to the community clinics only for urgent cases. All that may have led to a strong fear of getting close to a hospital, whereas there were people who got the virus and the medical staff came in close contact with these infected people.

 Another potential driver of this change might have been how the social networks (which were not developed 11 years ago) played a role in this pandemic compared to the previous ones. While the MoH in Israel instructed citizens to maintain social remoteness and isolation, we believe that other social networks had caused panic among the population, as Ahmad et al had shown in a study conducted among 516 social media users, during the COVID-19 outbreak.^14^

 A piece of evidence for the possible effect of the media on this behavior can be found when considering the behavior of the people in Israel during the 2006 Israeli-Lebanese war. During 30 days of the war, the hospital and its major surroundings were under missile attacks daily. During this period, the total number of visits to the ED dropped by 36% in comparison to the same period a year before (5794 vs. 9074) and the number of patients admitted in the hospital dropped by 34% (1084 vs. 1636) respectively.^15^ The Israeli media reported on these attacks constantly in addition to describing the general location where the missiles descend, pictures and videos of the consequences of these attacks. In a certain way, they showed a severe risk in visiting the hospital.

 During the COVID-19, 9.5% of the oncologic patients in our hospital postponed their urgent visits to the hospital, despite phone calls to urge them to come and have their chemotherapy. Furthermore, according to the Magen David Adom national ambulance report, there was a rise in 22% of at-home mortality during March 2020 in comparison to the same period a year before (1115 vs. 909).^16^ The data may support the notion that some might have endangered themselves as they avoided visiting the EDs for urgent cases. This may also explain the reduction in the number of myocardial infarctions during the COVID-19 outbreak period as was recently reported.^17^

 The decrease in the number of EDs referrals made hospitals to use TV news at the beginning of May 2020 to encourage people to go to the EDs, by emphasizing them as safe places free of COVID-19 infected patients. We assume that if both the MoH and the hospital spokespersons had acted earlier, people’s fear of visiting the EDs would have lessened.

 In conclusion, during this pandemic, the government leaders and the MoH used the official media, like the TV and the radio, in a different way, compared to previous pandemics. Again, social media, which was not developed 11 years ago, might have played some role in the population’s change of the behavior regarding the decrease in the number of visits to the EDs. The MoH had adopted fear as a strategy and advised citizens to visit community clinics only for urgent reasons. This might have influenced citizens to think that the community clinics and the hospitals are contagious places. Therefore, it seems appropriate to ask again, as did Srinivasan during the H1N1 pandemic: Is panic the key to successful modern health policy?^12^

 Whereas there may be value in causing people to fear a pandemic and by that causing them to behave in a way that would minimize their chances of getting sick, an extreme dose of fear seems to be more harmful than achieving its desired goal. Therefore, it would be better if our leaders emphasized the safety of hospitals alongside taking precautions in daily life to prevent the spread of the epidemic.

## Competing interests

 Authors declare that they have no competing interests.

## Ethical issues

 Since no personal information was disclosed and data was not collected for the purpose of the study but rather was taken from the hospital’s records, no ethical concerns about disclosure of information and other relevant issues are relevant.

## Authors’ contributions

 FB: preparation of data, literary review and write-up. HZ and KH: provision of data and help with literary review. SH: Data analysis and write-up.

## Authors’ affiliations

^1^Technion Faculty of Medicine, Rambam Health Care Campus, Haifa, Israel. ^2^Haifa University, Haifa, Israel.
